# Consistent high concentration of the viral microRNA BART17 in plasma samples from nasopharyngeal carcinoma patients - evidence of non-exosomal transport

**DOI:** 10.1186/1743-422X-10-119

**Published:** 2013-04-16

**Authors:** Claire Gourzones, François-Régis Ferrand, Corinne Amiel, Benjamin Vérillaud, Ana Barat, Maryse Guérin, Charles-Henry Gattolliat, Aurore Gelin, Jihène Klibi, Arij Ben Chaaben, Véronique Schneider, Fethi Guemira, Joël Guigay, Philippe Lang, Anne-Sophie Jimenez-Pailhes, Pierre Busson

**Affiliations:** 1 Université Paris-Sud 11, CNRS-UMR 8126 and Institut de Cancérologie Gustave Roussy, 39 rue Camille Desmoulins, Villejuif 94805, France; 2 Ecole du Val de Grâce, 1 place Alphonse Laveran, Paris 75005, France; 3 Virology Department, Hôpital Tenon, 4 rue de la Chine, Paris 75020, France; 4 Head and Neck Department, Hôpital Lariboisière, 2 rue Ambroise Paré, Paris 75010, France; 5 INSERM UMRS 939 - Université Pierre et Marie Curie - Paris6, Hôpital de la Pitié, 83 boulevard de l’Hôpital, Paris 75013, France; 6 Clinical Biology Department, Institut Salah Azaiz, Tunis, Tunisia; 7 Head and Neck Tumors Department, Institut de Cancérologie Gustave Roussy, 39 rue Camille Desmoulins, Villejuif 94805, France; 8 Radiotherapy Department, Hôpital Pitié-Salpétrière, 47 Boulevard de l’hôpital, Paris 75013, France

**Keywords:** Biomarker, DNA load, Epstein-Barr virus, Exosomes, Head and Neck carcinomas, Lipoproteins, miR-BART, microRNA, Nasopharyngeal carcinoma, Plasma

## Abstract

**Background:**

Because latent Epstein Barr (EBV)-infection is a specific characteristic of malignant nasopharyngeal carcinoma (NPC), various molecules of viral origin are obvious candidate biomarkers in this disease. In a previous study, we could show in a few clinical samples that it was possible to detect a category of EBV microRNAs called miR-BARTs in the plasma of at least a fraction of NPC patients. The first aim of the present study was to investigate the status of circulating miR-BART17-5p (one of the miR-BARTs hereafter called miR-BART17) and EBV DNA in a larger series of NPC plasma samples. The second aim was to determine whether or not circulating miR-BART17 was carried by plasma exosomes.

**Patients and methods:**

Plasma samples were collected from 26 NPC patients and 10 control donors, including 9 patients with non-NPC Head and Neck squamous cell carcinoma and one healthy EBV carrier. Concentrations of miR-BART17 and two cellular microRNAs (hsa-miR-16 and -146a) were assessed by real-time quantitative PCR with spike-in normalization and absolute quantification. In addition, for 2 patients, exosome distributions of miR-BART17 and miR-16 were investigated following plasma lipoprotein fractionation by isopycnic density gradient ultrcentrifugation.

**Results:**

The miR-BART17 was significantly more abundant in plasma samples from NPC patients compared to non-NPC donors. Above a threshold of 506 copies/mL, detection of miR-BART17 was highly specific for NPC patients (ROC curve analysis: AUC=0.87 with true positive rate = 0.77, false positive rate = 0.10). In this relatively small series, the concentration of plasma miR-BART17 and the plasma EBV DNA load were not correlated. When plasma samples were fractionated, miR-BART17 co-purified with a protein-rich fraction but not with exosomes.

**Conclusions:**

Detection of high concentrations of plasma miR-BART17 is consistent in NPC patients. This parameter is, at least in part, independent of the viral DNA load. Circulating miR-BART17 does not co-purify with exosomes.

## Background

Nasopharyngeal carcinoma (NPC) is a tumor arising from the epithelium lining the upper part of the pharynx behind the nasal cavities. Its incidence is very high in some highly populated regions of the world, especially Southeast Asia and North Africa [1]. This disease is responsible for the death of 50 000 people each year and hundred others suffer from the consequences of radiotherapy and chemotherapy. Therefore, in these countries, NPC is a major public health problem. In addition, NPC has become relatively frequent in some places of Europe and North America due to large numbers of oversea immigrants from endemic regions. Major issues to optimize the treatment of this disease are first, to lessen the rate of advanced disease at diagnosis by improving screening tools and policies, secondly, to personalize the treatment and follow up by identifying prognostic or predictive biomarkers.

One remarkable particularity of this disease is its constant association with the Epstein-Barr virus. EBV is one etiological factor of NPC which acts in combination with some forms of genetic susceptibility and/or environmental aggressions. The EBV genome is present in the nuclei of all malignant cells which are in their overwhelming majority latently infected. Despite the absence of viral replication, several viral genes are active in latently infected cells, driving production of a few viral proteins and non coding RNAs, including viral microRNAs. In EBV-infected cells, depending on the host cell background, EBV microRNAs can be transcribed from 3 clusters of viral genes, one located in the BamH1-H region (the BHRF1 cluster) and two located in the BamH1-A region (the BART clusters). In most NPC tumors, the BART clusters are transcribed at a very high level whereas the BHRF1 cluster is silent [2-5].

In a previous report, we have shown that BART miRNAs are secreted by NPC cells *in vitro* and are selectively detected in plasma samples from mice xenografted with NPC tumors but not in plasma samples from mice xenografted with non-NPC, EBV-negative tumors [6]. We have also reported the detection of miR-BART7 in a small series of plasma samples from NPC patients. These data suggested that circulating BART microRNAs could become a novel source of viral biomarkers for NPC population screening and patient monitoring. However, in this small set of patients, the average concentration of miR-BART7 was only moderately increased in the plasma of NPC patients by comparison with non-NPC controls, either healthy EBV-carriers or patients affected by non-NPC tumors. More recently, Wong et al. have confirmed the consistent detection of BART microRNAs in serum samples from NPC patients suggesting a higher specificity of detection for a sub-group of miR-BARTs including miR-BART17-5p (hereafter called miR-BART17) [7].

Therefore, we started this novel study with two aims: 1) to substantiate the notion that miR-BART17 is detectable with high specificity in plasma samples from NPC patients of various geographic origins and 2) to better characterize the vesicular or non-vesicular carriers of miR-BART17 in human plasma. We report that miR-BART17 is detected at a significantly higher concentration in the plasma of NPC patients by comparison with non-NPC donors and that its concentration is apparently not correlated to the EBV DNA load. In addition, we provide substantial evidence that circulating miR-BART17 molecules are associated with a protein-rich fraction of the plasma but not with circulating exosomes.

## Results

### High concentrations of miR-BART17 in plasma samples from NPC patients

**Figure 1:**
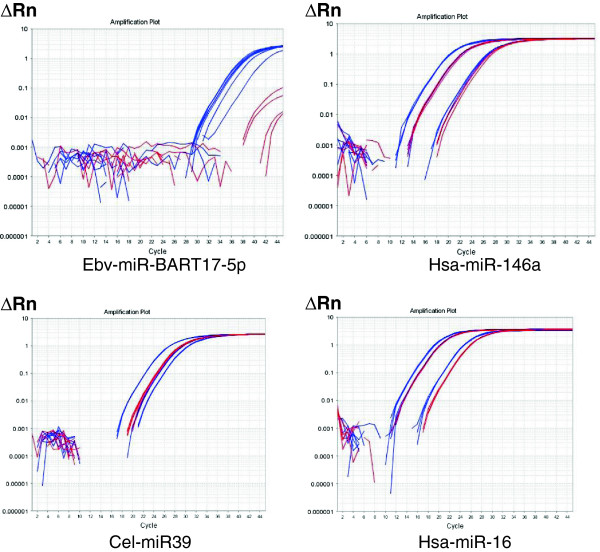
**Specific amplification of miR-BART17 in plasma RNAs from NPC patients (pilot study).** Concentrations of miR-BART17, miR-146a, miR-16 and cel-miR-39 microRNAs were assessed by qRT-PCR in plasma RNAs from 3 NPC patients (8, 16, 17; blue) and 2 controls (patients C1, C2 bearing non-NPC Head and Neck carcinomas; red). ΔRn stands for the magnitude of the fluorescent signal generated during the PCR at each time point (with background correction). Hsa-miR-16 and hsa-miR-146a are two cellular microRNAs known to be abundant in most human plasma samples. Cel-miR-39 is a *Caenorhabditis Elegans* microRNA without homology to mammalian microRNAs. A constant amount of a synthetic cel-miR-39 microRNA was added to each plasma sample at an early stage of the RNA extraction procedure, providing an exogenous control in order to detect RNA extraction bias. Amplification curves for cel-miR-39 are homogeneous in all 5 samples confirming the quality of the RNA extraction procedures. In contrast, the amplification curves are heterogeneous for hsa-miR-16 and has-miR-146 but without separation of samples from NPC patients and from control donors. On the contrary, marked amplifications of miR-BART17 are only seen for samples from NPC patients.

**Figure 2:**
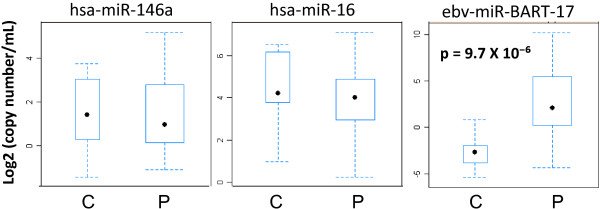
**Comparative distribution of miR-146a, miR-16 and miR-BART17 in plasma samples from NPC patients and control donors.** Samples from 26 NPC patients were compared to samples from 10 controls including 9 patients bearing non-NPC Head and Neck carcinomas and one healthy carrier. As shown in this Box Plot representation, micro-RNA concentrations are significantly different between NPC patients (P) and control donors (C) only for miR-BART17 (Wilcoxon test). Error bars refer to standard deviations.

**Figure 3:**
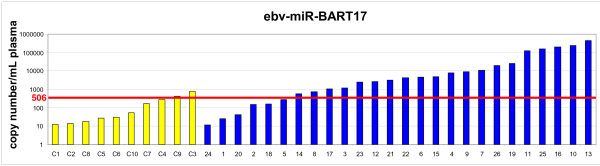
**Analysis of miR-BART17 distribution in plasma samples from NPC patients and control donors.** Histogram distribution of miR-BART17 copy numbers among NPC patients (blue bars) and controls (yellow bars) with their relation to the threshold of 506 copies/mL calculated by ROC analysis (see Additionnal file 2: Figure S1).

We first evaluated by RT-qPCR ebv-miR-BART17-5p (miR-BART17) and two cellular microRNAs - hsa-miR-146a and hsa-miR-16 - in a pilot series of plasma samples from 3 NPC patients and 2 control donors bearing non-NPC tumors (Figure 1). A synthetic microRNA from *Caenorhabditis Elegans*, cel-miR-39, was used as an exogenous control (it has no homology with mammalian microRNAs). The same amount of cel-miR-39 was “spiked-in” in all plasma samples at an early stage of RNA extraction in order to monitor RNA extraction bias. Hsa-miR-146a and hsa-miR-16 were used as endogenous references. They are known to be abundant in plasma samples from most human subjects although with wide inter-individual variations [8]. As shown in Figure 1, cel-miR-39 amplifications were homogeneous in all five plasma samples, reflecting the good quality of RNA extraction. As expected, amplifications of miR-146a and miR-16 were heterogeneous but without specific distribution for the samples from NPC patients or control donors. In contrast, significant amplifications of miR-BART17 were only seen for NPC patients. Therefore, we decided to extend this series to 27 consecutive NPC patients and 10 control donors (9 donors bearing non-NPC tumors and 1 healthy EBV-carrier). NPC patients were of various ethnical origins with a majority from North Africa. They presented either with localized (from stage II to stage IV b; UICC 2009) or metastatic disease at first occurrence or following a tumor relapse (Table 1). Some NPC patients had treatment prior to plasma sample collection but all of them had active disease at the time of blood drawing. Average CT values of the endogenous viral and cellular microRNAs (miR-BART17, miR-16 and miR-146) were normalized according to the CT values obtained for the exogenous control microRNA (miR-cel-39) in order to avoid RNA extraction bias (one NPC patient was thus excluded from the beginning because of an unexplained and repeated RNA extraction failure). Finally, absolute copy numbers of the endogenous microRNAs were deduced from calibration curves made with the corresponding synthetic purified microRNAs. Plasma concentrations of miR-BART17 are presented in Tables 1 (NPC patients) and 2 (control patients) along with patients characteristics. Concentrations of other circulating microRNAs are presented in Additional file 1: Table S1. It is noteworthy that the range of circulating microRNA concentrations (copy number/mL) was lower by several orders of magnitude for miR-BART17 compared to the cellular microRNAs. In summary, concentrations were in a range of 1 X 10^1^ to 6 X 10^5^ for miR-BART17 in contrast to a range of 5 X 10^5^ to 3 X 10^9^ for miR-16 and miR-146 (Additional file 1: Table S1). The median concentration of plasma miR-BART17 was about 100 times higher for NPC patients (3220 copies/ml) than for non-NPC donors (30 copies/ml) (p<0.0001) (Tables 1 and 2) (Figure 2). For optimal separation of NPC patients from control donors, a threshold of 506 copy/mL of plasma was determined by a ROC curve analysis (AUC= 0.87) providing a true positive rate (sensitivity) of 0.77 and a false positive rate (1-specificity) of 0.10 (Figure 3 and Additional file 2: Figure S1).

**Table 1:** Clinico-pathological characteristics, viral DNA load and plasma concentration of miR-BART17 normalized with miR-cel-39 for samples from NPC patients

**Patient**	**Gender/age**	**Origin**	**First occurrence (FO) or Relapse(R)**	**TNM**	**Histology**	**EBV DNA copy number/ml**	**miR BART17 copy number/ml**
1	M/59	France	FO	cT4N2M0	NKUC	1559	26
2	M/43	Morocco	R	rT0N0M1	NKUC	7065	148
3	M/55	Cambodia	FO	cT3N0M0	NKUC	28	1226
4	M/40	Algeria	FO	cT4N1M0	NKUC	325	8014
5	M/61	Algeria	FO	cT2N2M0	NKUC	466	279
6	M/47	China	FO	cT2N0M0	NKUC	0	4501
7	F/34	Sweden	FO	cT2N3aM1	NKUC	67	11087
8	F/47	Tunisia	FO	cT2N1M0	NKDC	0	739
9	M/40	North Africa	FO	cT1N3bM0	NKUC	40	8824
10	M/51	North Africa	FO	cT3N2M1	NKUC	1142	242506
11	M/41	Turkey	R	rTXN1M0	KSCC	79	128426
12	M/49	France	FO	cT3N1M0	NKUC	0	2725
13	M/65	France	R	rT2N3bM0	NKUC	0	448961
14	M/58	Tunisia	FO^*^	cT2N0M0	NKUC	351	589
15	M/41	Tunisia	FO^*^	cT3N2M0	NKUC	1475	4889
16	F/49	France	FO	cT2N2M0	NKUC	9	205985
17	F/39	Morocco	FO	cT4N1M0	NKUC	0	1080
18	M/62	Algeria	R^*^	rT4N0M0	NKUC	328	161
19	M/66	Algeria	R	rT0N2M0	NKUC	31	25788
20	M/47	Cameroon	R	rTXN2M1	NKUC	21	42
21	M/58	Cameroon	R^*^	cT2bN1M1	NKUC	8	3220
22	M/60	Morocco	R	rT1N2M0	NKUC	136	4401
23	M/59	Algeria	R	rT1NxM0	NKUC	0	2465
24	M/58	Algeria	FO^*^	cT1N3bM0	NKUC	20	11
25	F/40	France	R	rT0N0M1	NKUC	0	155002
26	M/61	Cameroon	R^*^	rT4N0M0	NKUC	0	19881

Samples were collected prior to any treatment (FO), without any treatment in the previous last 6 months (R) or from patients under chemotherapy or chemo-radiotherapy (FO* and R*). All patients had active disease at the time of blood sample collection. NKUC: non keratinizing undifferentiated carcinoma; NKDC: non-keratinizing differentiated carcinoma; KSCC: keratinizing squamous cell carcinoma.

**Table 2:** Clinico-pathological characteristics, viral DNA load and plasma concentration of miR-BART17 normalized with miR-cel-39 for samples from control donors

**Control donors**	**Gender/age**	**Origin**	**Primary tumor**	**TNM**	**Histology**	**EBV DNA copy number/ml plasma**	**miR BART17 copy number/ml plasma**
C1	M/54	Tunisia	larynx	T3N0M0	SCC	0	12
C2	M/53	Tunisia	larynx	T2N0M0	SCC	0	14
C3	M/59	France	larynx	T3N0M0	SCC	0	783
C4	F/60	France	larynx	T2N0M0	SCC	0	277
C5	M/55	France	larynx	T3N0M0	SCC	0	27
C6	M/448	France	hypopharynx	T3N0M0	SCC	0	30
C7	M/47	France	larynx	T3N0M0	SCC	0	174
C8	M/56	France	no tumor	na	na	0	18
C9	F/226	France	maxilary sinus	T4N1M0	SCC	0	435
C10	M/52	France	hypopharynx	T3N2M0	SCC	0	54

SCC: squamous cell carcinoma. C8 was a healthy donor. All other plasma samples except C10 were collected prior to treatment from patients bearing Head and Neck non-NPC carcinomas. The sample from patient C10 was collected under therapy.

### Apparent lack of correlations between miR-BART17 concentrations and EBV-DNA loads in NPC plasma samples

**Figure 4:**
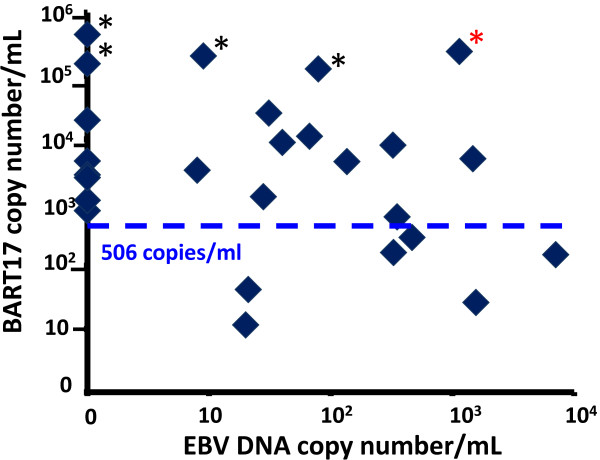
**Comparative distribution of plasmatic EBV DNA load and miR-BART17 copy numbers among NPC patients.** The numbers on the axes are for the EBV DNA (x axis) and miR-BART17 (y axis) copies per mL of plasma (logarithmic scales). The samples from each NPC patient appear as diamond-shaped dots. The horizontal dotted line represents the threshold of 506 copies per mL defined by ROC analysis (see the legend of Figure 3). Five patients (10, 11, 13, 16 and 25) have more than 10^5^ copies of miR-BART17 per mL (dots with asterisks). Only one of them (patient 10) has an EBV DNA load with more than 10^2^ copies/mL (one dot on the upper right of the graph marked with a red asterisk).

We found no apparent relationships between the plasma concentrations of miR-BART17 and the plasma EBV DNA load (Figure 4). It is noteworthy that among 5 NPC patients with more than 10^5^ copies of miR-BART17 per mL, four had less than 200 copies of EBV-DNA per mL. Our series had obviously too much heterogeneity to allow tentative correlations between clinical parameters and miR-BART17 or EBV DNA copy numbers in plasma samples.

### Changes in plasma concentrations of miR-BART17 according to tumor evolution in one patient

**Figure 5:**
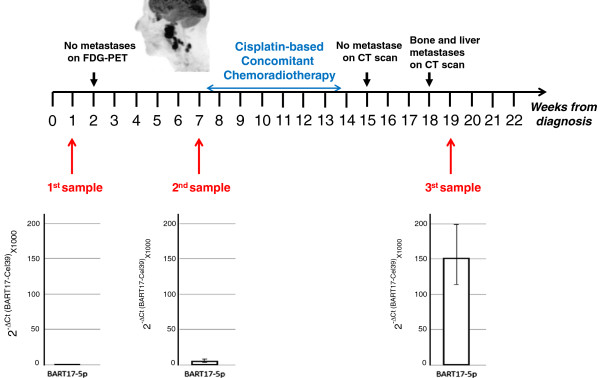
**Evolution of miR-BART17 concentrations in plasma samples from an NPC patient with rapid disease progression.** Several plasma samples were sequentially collected from the NPC patient n°5. Two of them were obtained prior to any treatment. The third one was obtained 5 weeks after completion of chemoradiotherapy in a context of very rapid, quasi-immediate, metastatic relapse. This tumor was initally localized (T2N2M0) but it was highly hypermetabolic as shown on the sagittal image of ^18^Fluoro Deoxyglucose Positron Emission Tomography (FDG-PET) combined to the computed tomography (CT) scan (SUVmax = 30.9). Data from miR-BART17 PCR-amplification were normalized using data from concomitant cel-miR-39 amplification. Relative amounts of miR-BART17 were calculated according to the 2^-ΔCt^ method. Error bars refer to standard deviations. In the final sample collected in the context of the metastatic relapse, the concentration of plasma miR-BART17 was more than 100 times higher than in the initial sample.

One patient presented in Table 1 (NPC patient n°5) initially classified as T2N2M0 experienced a dramatic tumor progression under therapy with rapidly growing nodal and distant metastases (Figure 5). We could obtain 3 sequential plasma samples from this patient. (1, 7 and 19 weeks following the time of the diagnosis). Real time PCR analyses were performed simultaneously on these 3 samples demonstrating an exponential increase of miR-BART17 plasma concentrations which accompanied the rapid expansion of tumor lesions. There was a concomitant increase in the EBV DNA load from 2 555 to 466 396 copies/mL from week 7 to 19.

### Distribution of miR-BART17 and hsa-miR-16 among plasma lipoproteins separated by isopycnic density gradient ultracentrifugation

**Figure 6:**
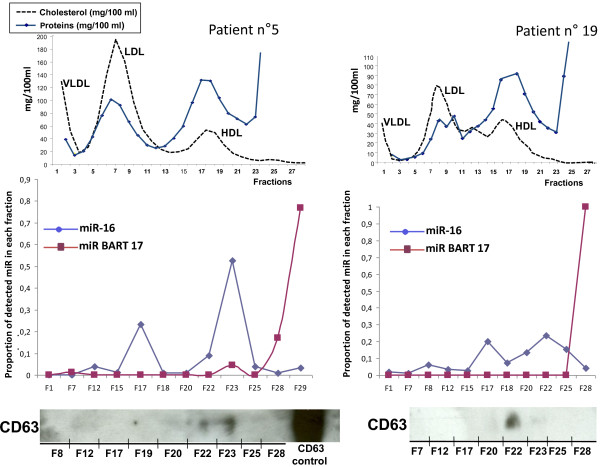
**Distribution of miR-BART 17 in plasma fractions obtained by flotation on a potassium bromide gradient.** Three mL plasma aliquots from 2 NPC patients (n°5 and 19) were fractionated as described in the Materials and Methods section. Top panels. Twenty nine fractions were analyzed for their cholesterol (dotted black line) and protein (solid blue line) contents. From the top to the bottom of the gradient, three major cholesterol peaks allow identification of fractions containing VLDL (<1.006 g/mL), LDL (1.019 to 1.063 g/mL) and HDL (1.06 to 1.13 g/mL). Protein concentration was maximal at the bottom of the gradient. Within fractions 25 to 28, it goes above 1000 mg/100mL. Middle panels. Concentrations of miR-16, miR-BART17 and cel-miR-39 were determined in 11 to 12 selected fractions evenly distributed through the gradient. They are presented as the ratio of the number of microRNA copies contained in each fraction to the sum of the copies contained in all fractions. MiR-16 is mainly distributed in 2 peaks: one small peak coincident with fraction 17 and one bigger peak coincident with the portion of the gradient containing the exosomes. In contrast, miR-BART17 is recovered almost exclusively in the bottom fraction. Bottom panels. Detection of the CD63 protein by Western blot analysis in a set of selected fractions. After long exposure, CD63 signals are detected in fractions 22 and 23 (NPC patient n°5, left panel) and fraction 22 (NPC patient n°19, right panel). The densities of these fractions are in the range classically attributed to exosomes (1.15 to 1.19 g/mL).

As shown in the Additional file 1: Table S1, concentrations of plasma miR-BART17 are very low by comparison with the circulating cellular microRNAs, miR-16 and miR-146a. In order to increase the sensitivity and specificity of miR-BART17 detection, we attempted to identify specific carrier elements of this microRNA in human plasma samples. According to previous reports, plasma microRNAs can be associated with at least 4 types of plasma carriers: microvesicles, exosomes, high density lipoproteins (HDL) and non-vesicular ribonucleoprotein complexes often containing the argonaute 2 protein [8-11]. Therefore, to get clues about the relationships between BART microRNAs and these various types of potential plasma carriers, we chose an open experimental approach allowing simultaneous assessment of these various carriers, i.e. flotation of plasma elements on a KBr gradient. This method is commonly used for separation of plasma lipoproteins and is also appropriate for exosome separation [9,10]. Plasma samples from 2 NPC patients were fractionated on KBr gradients. RNAs were purified from several fractions evenly distributed through the gradient (11 to 12 out of 29). Then miR-16 and miR-BART17 were quantified by qRT-PCR. Simultaneously, the fractions corresponding to the various types of lipoproteins were identified by assessment of cholesterol concentration in each fraction. Surprisingly, the cellular and the viral microRNAs did not co-purify with the same fractions (Figure 6). The miR-16 was recovered in two peaks; one small peak centered on fraction 17 and one bigger peak centered on fraction 22 (NPC patient n°19) or 23 (NPC patient n°5). In contrast, miR-BART17 was detectable almost exclusively in the fractions 28 and 29, where most proteins were recovered, with the exception of a very small amount detected in fraction 23 for one donor (NPC patient n°5). Exosomes are known to have a density of 1.15 g/mL to 1.19 g/mL and are expected to float deeper than the HDL peak (1.06 to 1.13 g/mL) [9]. To confirm the identity of the fractions containing the exosomes, several bottom fractions of the gradients were analyzed by Western blot for detection of CD63, a classical exosome marker. Consistent with the known density of exosomes most of the CD63 protein was detected in fractions 22 and/or 23, very close to the highest peak of miR-16, indicating that at least a fraction of miR-16 co-purified with exosomes in contrast to miR-BART17.

## Discussion

Investigations of circulating microRNAs in malignant diseases is currently a very active field and a large amount of data have already been published about this subject [8,11-13]. One remarkable characteristic of circulating microRNAs is their stability. To a large extent, it results from their association with various types of carriers. Some of these carriers are vesicular with a relatively large size; the two main categories are microvesicles (100 nm to 1 μm in diameter) and exosomes (30 to 100 nm). There are also non-vesicular carriers of smaller size like the HDL lipoproteins and non-lipid ribonucleoprotein complexes which, to a large extent, remain to be characterized [10,11,14,15].

NPC appears as a privileged model for investigations of circulating tumor microRNAs for two reasons: 1) malignant cells are latently infected by EBV in virtually all NPCs; 2) NPC cells have intense production of microRNAs from the BART cluster in the absence of production from the BHRF1 cluster [2,4,16]. It is not yet clear whether mir-BARTs can be produced in the healthy EBV-carrier or outside tumor tissues in NPC patients. According to *in vitro* models, latently infected B-cells are not expected to produce miR-BARTs but rather BHRF1 microRNAs [2,4,17]. It is known that the EBV lytic-replicative cycle is consistently taking place in the epithelial cells of the oral cavity (including tonsils and may be salivary glands) [18]. We do not know yet whether these lytically infected epithelial cells produce and release EBV miR-BARTs.

Our data were obtained from NPC plasma samples mainly collected from patients born in France and North Africa. They are consistent with the data reported by Wong et al. in a series of serum samples from 15 Chinese patients [7]. Presence of circulating miR-BART17 thus appears as a consistent feature of the disease, regardless of patient origins. Although there are discussions on whether use of serum or plasma samples is optimal for recovery of circulating microRNAs, our detection of miR-BART17 from plasma samples was apparently as efficient as the detection reported by Wong et al. who worked with serum samples [7,19,20]. In contrast with Wong et al., we could detect small amounts of miR-BART17 in plasma samples from non-NPC donors. This might be due to the fact that we took in account samples with very small concentrations of microRNAs, namely those giving Ct values higher than 35, whereas they are automatically classified as negative by many investigators. The presence of miR-BART17 in plasma samples from a few control donors might reflect low-level production of miR-BARTs by non-malignant cells for example in the oral cavity. This should not undermine the main conclusion of our study: detection of the miR-BART17-5p above the threshold of 506 copies/mL appears as a marker of NPC plasma samples with good sensitivity (77%) and high specificity (90%).

To further improve the sensitivity and specificity of miR-BART detection in plasma samples, we attempted to characterize their carriers. On the basis of previous results, we were assuming that there were carried by NPC tumor exosomes [6]. However, fractionation of plasma elements on a KBr gradient showed that the cellular micro-RNA, miR-16, partially co-purified with exosomes whereas miR-BART17 was recovered in a completely distinct fraction. Although it has only been observed for our two samples subjected to gradient fractionation, this observation may be relevant to the design of future studies. On the one hand, the lack of co-purification of miR-BART17 with exosomes is surprising since we and others have shown that the BART microRNAs are abundant in exosomes released by EBV-infected cells *in vitro*[6,21]. On the other hand, it appears to be consistent with a recent report about liver microRNAs [22]. This report shows that, depending on physiological or pathological conditions, the same micro-RNAs co-purify either with an exosome-rich or a protein-rich fraction of the plasma. Therefore we should consider the hypothesis that the miR-BARTs are secreted in association with exosomes by NPC cells *in vitro* but not in the tumor context *in vivo*. The concentration of miR-BART17 at the bottom of the KBr gradient suggests that it is associated with non-floating elements, probably ribonucleoprotein complexes of small size and/or devoid of lipids which remained to be characterized. Other groups have reported incorporation of plasma microRNAs in non-vesicular complexes containing the Agonaute 2 (ago2) protein [14]. In future investigations, it will be interesting to know whether plasma ribonucleoprotein complexes containing miR-BART17 can be immunoprecipitated with anti-ago2 antibodies.

Our series of NPC patients was obviously too heterogeneous to attempt correlations between the concentration of plasma miR-BART17 and clinical parameters. However, we were able to collect sequential plasma samples for one patient (Patient n°5) who had a rapidly progressive disease. We observed a dramatic increase in the concentration of miR-BART17 which was parallel to tumor progression. This suggests that the plasma concentration of miR-BART17 is related, at least in part, to the tumor mass. That point will require further investigations. In this small series, we have found no obvious correlations between the concentrations of plasma miR-BART17 and the plasma EBV DNA loads. One can speculate that both miR-BART and EBV DNA plasma copy numbers are affected by multiple factors: the total tumor burden, the apoptotic index of the malignant cells, their rate of necrosis, the vascularization of tumor lesions and the rate of degradation of microRNAs and DNA by peripheral nucleases. However, the relative impact of each of these factors is probably different for the miR-BARTs and for the EBV-DNA. This could explain why their plasma concentrations might evolve independently at various stages of the disease. On the other hand, if the concentrations of miR-BARTs and EBV DNA are at least partially independent, they have more chances to provide non-redundant information for assessment of the tumor status and/or prediction of the tumor evolution. In order to substantiate this hypothesis, we currently investigate: 1) the relationships between the abundance of circulating miR-BARTs and the total tumor mass in a series of non-metastatic NPC patients; 2) the longitudinal evolution of circulating miR-BARTs in connection with the tumor response to the initial treatment. While this study was near completion, Chan et al. have reported a consistent detection of miR-BART7 in the plasma of NPC patients [23]. Both studies converge on two important points: 1) EBV BART microRNAs are detectable in the plasma of some non-NPC donors but at a low level in contrast with most NPC patients; 2) the concentration of circulating miR-BARTs seems to be relatively independent of the plasma viral DNA load.

## Conclusions

This study confirms the consistent and specific presence of a circulating miR-BART in plasma samples from NPC patients. We provide evidence that the concentration of plasma miR-BART17 is, at least in part, independent of the plasma viral DNA load. In addition, we show that this microRNA is not bound to plasma exosomes but probably associated to non-vesicular ribonucleoprotein complexes. These observations will provide a basis to address questions about the relationship of plasma miR-BARTs with NPC volumetric characteristics and about their possible role in the prediction of tumor response under treatment.

## Materials and methods

### Collection, separation and storage of human plasma samples

This study was performed with the approval of the Tarnier-Cochin Ethics Committee (project n° 2746 approved on January 27, 2010). Blood samples from 36 donors were collected after signature of written informed consent, from June 2010 to October 2011. Twenty six of these samples were collected from NPC patients admitted to the Institut de Cancérologie Gustave Roussy or Paris hospitals working in collaboration with this Institute (Table 1). All these patients had evidence of active disease, either as a first occurrence or as a recurrence. Blood samples were generally collected before initiation of the treatment (Table 1). Control samples were obtained from 9 consecutive patients admitted to the Department of Head and Neck Oncology at the Institut de Cancérologie Gustave Roussy for non-NPC tumors. All these tumors were squamous cell carcinomas of the upper aero-digestive track. More clinical details are provided in Table 2. One additional control plasma sample was obtained from a healthy EBV-carrier donor. For all donors, plasma was separated from blood collected in EDTA tubes by centrifugation at 1700 g at 20°C for 15 min, aliquoted and frozen at – 80°C. In virtually all cases, plasma separation and freezing was done in less than 2 hours following blood collection.

### Clinical staging and pathological diagnosis

Tumor staging was performed according to UICC 7^th^ edition (Union for International Cancer control) guidelines [24]. Histological classification was performed according to the 2005 WHO classification [25]. Except for 4 patients, the histological diagnosis was confirmed by detection of EBV products on tissue sections, either the LMP1 protein detected by immunohistochemistry (3 cases) or the EBERs (Epstein-Barr encoded RNAs) detected by *in situ* hybridization using commercial kits, mainly from Ventana Medical System (Illkirch, France) [26].

### Isolation and chemical analysis of plasma lipoprotein fractions

Plasma lipoproteins were isolated from 2 selected fasting plasma samples (2 to 3 mL) by density gradient ultracentrifugation in a Beckman SW41 Ti rotor at 40 000 rpm for 42 hours in a Beckman XL70 at 15°C as previously described [9]. Plasma density was increased to *d*=1.21 g/mL by addition of dry, solid KBr. A discontinuous density gradient was constructed as follows: 2 mL of NaCl-KBr solution of *d*=1.24 g/mL; 3 mL of plasma adjusted to a background density of *d*=1.21 g/mL; 2 mL of *d=*1.063 g/mL; 2.5 mL of *d*=1.019 g/mL; and finally, 2.5 mL of NaCl solution of *d*=1.006 g/mL. After centrifugation, gradients were collected from the top of the tubes with an Eppendorf precision pipette in 30 fractions corresponding to VLDL (density <1.006 g/mL, fraction number 1), IDL (density from 1.006 g/mL to 1.019 g/mL, fraction number 2), 10 LDL subfractions (density from 1.019 g/mL to 1.063 g/mL, fraction number 3 to 12) and 11 HDL subfractions (density from 1.063 g/mL to 1.179 g/mL, fraction number 13 to 23). Lipoprotein fraction number 13 to 18 corresponded to HDL2 fraction (density from 1.063 to 1.110 g/mL), lipoprotein fraction number 19 to 23 corresponded to HDL3 fraction (density from 1.110 to 1.133 g/mL), and gradient fraction numbers 24 to 30 mostly contain proteins. All fractions were dialyzed in Spectropor membrane tubing against Phosphate Buffer Saline at pH7.4 before analysis for their content in total cholesterol using kit reagents from Roche Diagnostics and in total protein using the bicinchoninic acid assay from Pierce.

### RNA purification from plasma samples and use of “spike-in” controls

Total RNA was purified using the miRVana PARIS kit (Ambion, Austin, TX) according to the manufacturer instructions for total RNA purification from biological fluids. At the beginning of the procedure, 300 μl of plasma were mixed with 300 μl of 2X denaturing buffer and then incubated for 10 minutes on ice. Just before the next step which consisted in acid phenol/chloroform extraction, we added to each sample a synthetic microRNA identical to the *C.Elegans* cel-miR-39 (7 fmol) (Eurofins MWG Operon, Ebersberg, Germany).

### RNA purification from fractions obtained after plasma fractionation on KBr gadients

One hundred μl of each fraction (11 to 12 fractions out of 29) were diluted in 200 μl of PBS 1X and then mixed with 300 μl of 2X denaturing buffer. Total RNA was then purified as described for crude plasma RNA.

### CD63 detection by Western Blot after plasma fractionation on KBr gradients

Four μg of protein were loaded on pre-casted, gradient Acryl/BisAcryl 4-20% SDS gels purchased from Pierce (Thermo Scientific, Brebières, France). Protein migration and separation were performed in non-reducing conditions to allow CD63 detection. CD63 was detected with a mouse monoclonal antibody (TS63) kindly provided by Eric Rubinstein [27].

### Determination of the EBV DNA load in plasma samples

Total DNA was extracted from 200 μl plasma aliquots using the QIAmp blood DNA Minikit (Qiagen Inc., Courtaboeuf, France). Viral load was then determined by real-time quantitative PCR using a commercial kit from Argene (EBV R-gene™; France). The copy number of EBV DNA per milliliter of plasma was determined using a calibration curve based on serial dilutions of a standard provided in the kit. To rule out DNA extraction bias, albumin DNA copies were quantified in parallel in the same extracts as previously described [28].

### MicroRNA amplification, detection and quantification by quantitative RT-PCR

Real time RT-PCR analysis of the microRNAs was performed using the Taqman MicroRNA Reverse Transcription Kit and the Taqman MicroRNA Assay (Applied Biosystems, Foster City, CA). For reverse transcription, 5.84 μl of a mix containing 3 μl of the RT primer solution (final concentration: 50 nM ), 0.15 μl dNTP (1 mM), 1 μl Multiscribe Reverse Transcriptase (3.33 U/μl), 1.50 μl of 10X Buffer and 0.19 μl RNase inhibitor (0.25 U/μl) were added to 9.16 μl of plasma RNA (final volume: 15 μl). The mix was then incubated successively at 16°C for 30 minutes, 42°C for 30 min and 85°C for 5 min. After reverse transcription, PCR amplification was performed in 20 μl containing 4 μl of the final Reverse Transcription reaction mix mixed with 10 μl of FastStart Universal Probe Master mix (Roche diagnostics, Basel, Switzerland), 1 μl of the primer mix including - for a given microRNA - the universal reverse primer (0.7 μM), the specific primer (1.5 μM) and the hydrolysis probe (0.2 μM) (Taqman microRNA assay, Applied Biosystems) and 5 μl of nuclease free water (Ambion, Austin, TX). The first cycle included one step of 2 min at 50°C and one step of 10 minutes at 95°C. It was followed by 45 cycles including one step of 15 sec at 95°C and one step of 60 sec at 60°C. Multiple negative water blanks were included in every analysis. Amplification was performed in a StepOnePlus Detection System (Applied Biosystems).

Absolute quantification of microRNAs was done by comparison with 10-fold serial dilutions of synthetic double-strand oligonucleotides containing the same sequences (Eurofins MWG Operon, Ebersberg, Germany for ebv-miR-BART17-5p and cel-miR-39 and miRVana miRNA reference Panel v9.1, Ambion for hsa-miR-16a and hsa-miR-146a). The concentration of the first dilution was 0.2 fmol/μl. The following sets of primers and probes were purchased from Applied Biosystems (TaqMan MicroRNA assays): ebv-miR-BART17-5p (assay ID 008216), hsa-miR-16 (000391), hsa-miR-146a (000468) and cel-miR-39 (000200). Calculation of the average “Concentration threshold” (Ct) values from the triplicate was normalized to cel-miR-39 according to the method described by Kroh et al. [29].

### Statistical analysis

Subjects were divided into a "condition" present group, including all patients with NPC and a "condition" absent group which included control subjects. Wilcoxon test was used to compare differences in plasma microRNA ratios between the NPC group and the control group. P-value < 0.05 was considered significant. Receiver-operating-characteristic (ROC) curves and the area under the ROC curve (AUC) were used to assess the feasibility of using plasma miRNAs as diagnostic tools for detecting NPC. The ROC curve, together with a good tradeoff between the true positive rate and the false positive rate was used to determine the cutoff value for the concentrations of plasma microRNAs. A centered and scaled Principal Component Analysis (PCA) was performed to assess variability and the correlations in the multivariate dataset containing clinicopathological characteristics of patients, plasma miRNAs (miR-146, miR-16, miR-BART17) and the viral load. All analyses were performed using the R software for statistical analysis (R version 2.14.1).

## Abbreviations

AUC: Area under curve; EBERs: Epstein-Barr encoded RNAs; EBV: Epstein Barr virus; HDL: High density lipoprotein; LDL: Low density lipoprotein; LMP1: Latent membrane protein1; NPC: Nasopharyngeal carcinoma; ROC: Receiver-operating-characteristic; UICC: Union for International Cancer control; VLDL: Very low density lipoprotein; WHO: World Health Organization.

## Competing interests

The authors declare that they have no competing interests.

## Authors’ contributions

CG and FRF made RNA and c-DNA preparations and PCR analyses. They were involved in the design of the study and preparation of the manuscript. FRF, JG, BV, PL, FG, JK and ABC participated in sample collection and their clinical annotation as well as separation and storage of several plasma samples. ASJ and AG participated in the separation and storage of the plasma samples and in RNA extraction. CHG shared his expertise for handling qPCR on microRNAs. CA and VS have assayed the viral DNA load in plasma samples. MG has done plasma fractionations on KBr gradients. AB participated in the statistical analysis. PB participated in the design of the study and its coordination and drafted the manuscript. All authors read and approved the final manuscript.

## Supplementary Material

Additional file 1: Table S1 EBV DNA and miR-copy numbers from plasma samples of NPC patients and controls. (1) UICC staging is specified only for patients with a first occurrence of NPC. (2) MicroRNA and DNA concentrations are expressed in copy number/mL. Click here for file

Additional file 2: Figure S1 Receiver-operating characteristic (ROC) curve analysis for miR-BART 17 copy numbers in plasma samples from 26 NPC patients (see Table 1) compared to 10 controls (see Table 2). From the ROC curve, the AUC (area under the curve) was calculated allowing determination of a cut-off value at 506 copies per mL with a sensitivity of 77% and a specificity of 90%. Click here for file
