# Supplementary material for: Consistent high concentration of the viral microRNA BART17 in plasma samples from nasopharyngeal carcinoma patients - evidence of non-exosomal transport
Source: Virol J. 2013 Apr 16;10:119. doi: 10.1186/1743-422X-10-119 (PMC3685608; doi:10.1186/1743-422X-10-119)
Supplement: Additional file 1: Table S1 — EBV DNA and miR-copy numbers from plasma samples of NPC patients and controls. (1) UICC staging is specified only for patients with a first occurrence of NPC. (2) MicroRNA and DNA concentrations are expressed in copy number/mL. [file 1743-422X-10-119-S1.doc]

**Supplementary table 1.** EBV DNA and miR-copy numbers from plasma samples of NPC patients and controls.

patient

TNM

UICC

Stage

ebv-miR-BART-17-5p

hsa-miR-146a

hsa-miR-16

DNA EBV

1

c T4N2M0

IVA

26

missing

107351386

1559

2

rT0N0M1

149

262714107

2502718031

7065

3

cT3N0M0

III

1226

42356614

275674202

28

4

c T4N1M0

IVA

8014

487425253

3057938090

325

5

cT2N2M0

III

279

3915940

356059546

466

6

cT2N0M0

II

4501

1593381

188946582

249

7

cT2N3aM1

IVC

11087

4169013

150358797

67

8

cT2N1M0

II

739

12498885

39020232

0

9

c T1N3bM0

IVB

8824

3000875

130999662

40

10

cT3N2M1

IVC

242506

58128002

1283928340

1142

11

rTXN1M0

128426

578241300

493639432

79

12

cT3N1M0

III

2725

10052459

8884655

0

13

rT2N3bM0

448961

6336372

149307108

0

14

cT2N0M0

II

589

88166979

134472115

351

15

cT3N2M0

III

4889

140733720

362709736

1475

16

cT2N2M0

III

205985

7196511

473886070

9

17

cT4N1M0

IVA

1080

13288539

359943000

0

18

rT4N0M0

161

59691796

476919607

328

19

r T0N2M0

25788

32569509

519161642

31

20

rTXN2M1

42

4935685

281900241

21

21

cT2bN1M1

3220

3111114

116710741

8

22

rT1N2M0

4401

1271946

62449626

136

23

rT1NxM0

2465

6132687

179772563

0

24

cT1N3bM0

IVB

12

593838

1211111

20

25

rT0N0 M1

155002

15785908

280285955

0

26

r T4N0M0

19881

2337517

73625404

0

c1

12

3392031

7464244

0

c2

14

111277826

286068005

0

c3

783

4892202

60180623

0

c4

277

57969948

1190803814

0

c5

27

616492

166096354

0

c6

30

10365142

4235456

0

c7

174

59005036

1387873383

0

c8

18

11148253

142602649

0

c9

435

24057998

1624797870

0

c10

54

3102654

199732519

0

(1) UICC staging is specified only for patients with a first occurrence of NPC.

(2) MicroRNA and DNA concentrations are expressed in copy number/mL
