# Supplementary material for: Consistent high concentration of the viral microRNA BART17 in plasma samples from nasopharyngeal carcinoma patients - evidence of non-exosomal transport
Source: Virol J. 2013 Apr 16;10:119. doi: 10.1186/1743-422X-10-119 (PMC3685608; doi:10.1186/1743-422X-10-119)
Supplement: Additional file 2: Figure S1 — Receiver-operating characteristic (ROC) curve analysis for miR-BART 17 copy numbers in plasma samples from 26 NPC patients (see Table 1) compared to 10 controls (see Table 2). From the ROC curve, the AUC (area under the curve) was calculated allowing determination of a cut-off value at 506 copies per mL with a sensitivity of 77% and a specificity of 90%. [file 1743-422X-10-119-S2.ppt]

## Slide 1
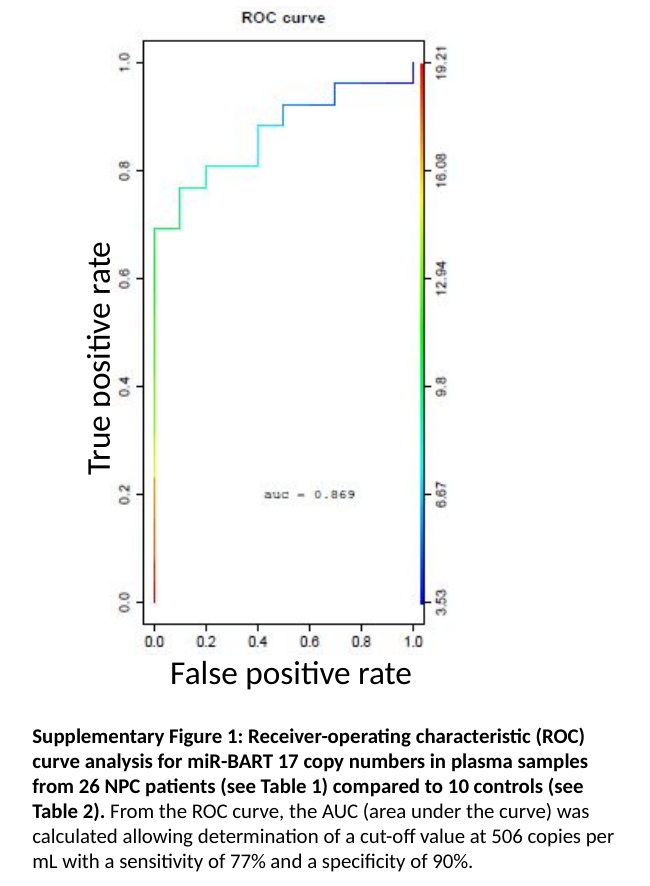

True positive rate
False positive rate
Supplementary Figure 1: Receiver-operating characteristic (ROC) curve analysis for miR-BART 17 copy numbers in plasma samples from 26 NPC patients (see Table 1) compared to 10 controls (see Table 2). From the ROC curve, the AUC (area under the curve) was calculated allowing determination of a cut-off value at 506 copies per mL with a sensitivity of 77% and a specificity of 90%.
